# Unveiling the complex networks of urban tree diversity research: A global perspective

**DOI:** 10.1002/ece3.11630

**Published:** 2024-06-21

**Authors:** Chunping Xie, Shuifei Chen, Dawei Liu, Chi Yung Jim

**Affiliations:** ^1^ Tropical Biodiversity and Bioresource Utilization Laboratory Qiongtai Normal University Haikou China; ^2^ Nanjing Institute of Environmental Sciences Ministry of Ecology and Environment Nanjing China; ^3^ Key Laboratory of State Forest and Grassland Administration Wildlife Evidence Technology Nanjing Police University Nanjing China; ^4^ Department of Social Sciences and Policy Studies Education University of Hong Kong Tai Po China

**Keywords:** bibliometric analysis, knowledge graph, research frontier, research hotspot, sustainable urban forest management, urban tree diversity

## Abstract

Ecosystem services offered by urban forests must be proactively managed to remain diverse and sustainable. Recent research findings deserve a systematic synthesis to elucidate inherent knowledge structures and dynamics. This study focused on the urban tree diversity theme from 2000 to 2022. Web of Science Core Collection database provided bibliometric details on academic publications. The data‐driven quantitative analysis explored research quantities, emphasis, trends, patterns, linkages, and impacts by countries, institutions, authors, journals, and citations. Publications and research topics have expanded continually, with accelerated growth in recent years. Research activities, outputs and interactions demonstrated conspicuous spatial clustering. A few countries, institutions and researchers generated a notable proportion of publications. Their scholarly contributions were visualized in knowledge graphs as complex networks of nodes and inter‐node links. Keyword analysis generated a network to indicate research hotspots and frontiers to steer and prioritize future studies. Recent findings affirmed that cities can harbor substantial tree diversity due to enhanced habitat heterogeneity and successful species adaptation. Aligning tree traits with environmental conditions and management objectives can improve benefits. Urbanization can filter tree traits to shape community assemblages through stressors: habitat degradation, fragmentation and loss, in conjunction with pollution, climate change, and introduced species. Diversity preservation strategies include protecting remnant natural vegetation, connecting green spaces, and restoring complex canopy geometry and biomass structure. The emerging frontiers are marked by modeling future species distributions, leveraging technologies like remote sensing, linking ecology with human values, and committing to community‐based stewardship. Management can be upgraded by interdisciplinary perspectives integrating ecological science and social engagement. The findings highlight the need for biodiversity enrichment anchored by native species, trait‐matched assemblages, adaptive policies, and community participation to create livable‐green cities. This review synthesizes key advances in urban tree ecology and biodiversity research to inform the planning and stewardship of resilient urban forests.

## Graphical abstract

This article summarizes a study that examined the research on urban tree diversity from 2000 to 2022. The study used bibliometric analysis to explore trends, patterns, and impacts of research activities in this field. The findings indicate that urban tree diversity research has been expanding, with a few countries, institutions, and researchers making significant contributions. The study also identifies research hotspots and frontiers, such as modeling future species distributions and integrating ecological science with social engagement, to guide future studies and improve the management of urban forests.

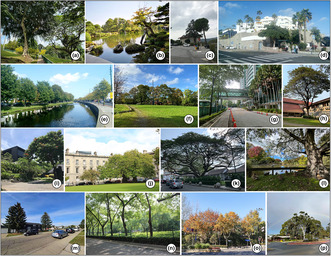

## 1 INTRODUCTION

Urban tree diversity is critical for sustaining multiple ecosystem services and benefits (Davies et al., 2017; Duinker et al., 2015). Trees in cities comprise diverse species with different origins, functional traits, and environmental adaptations (Esperon‐Rodriguez et al., 2020). Higher urban tree diversity enhances ecosystem functions, including carbon sequestration, temperature regulation, stormwater mitigation, pollution removal, and wildlife habitat (O'Sullivan et al., 2017). Diverse tree communities with greater genetic, taxonomic, and functional varieties have higher structural complexity and resiliency against environmental stressors like pests, pathogens, and climate fluctuations. In contrast, low‐diversity urban forests dominated by a few species are vulnerable to disease, insect outbreaks, and die‐offs (Raupp et al., 2006).

**FIGURE 1:**
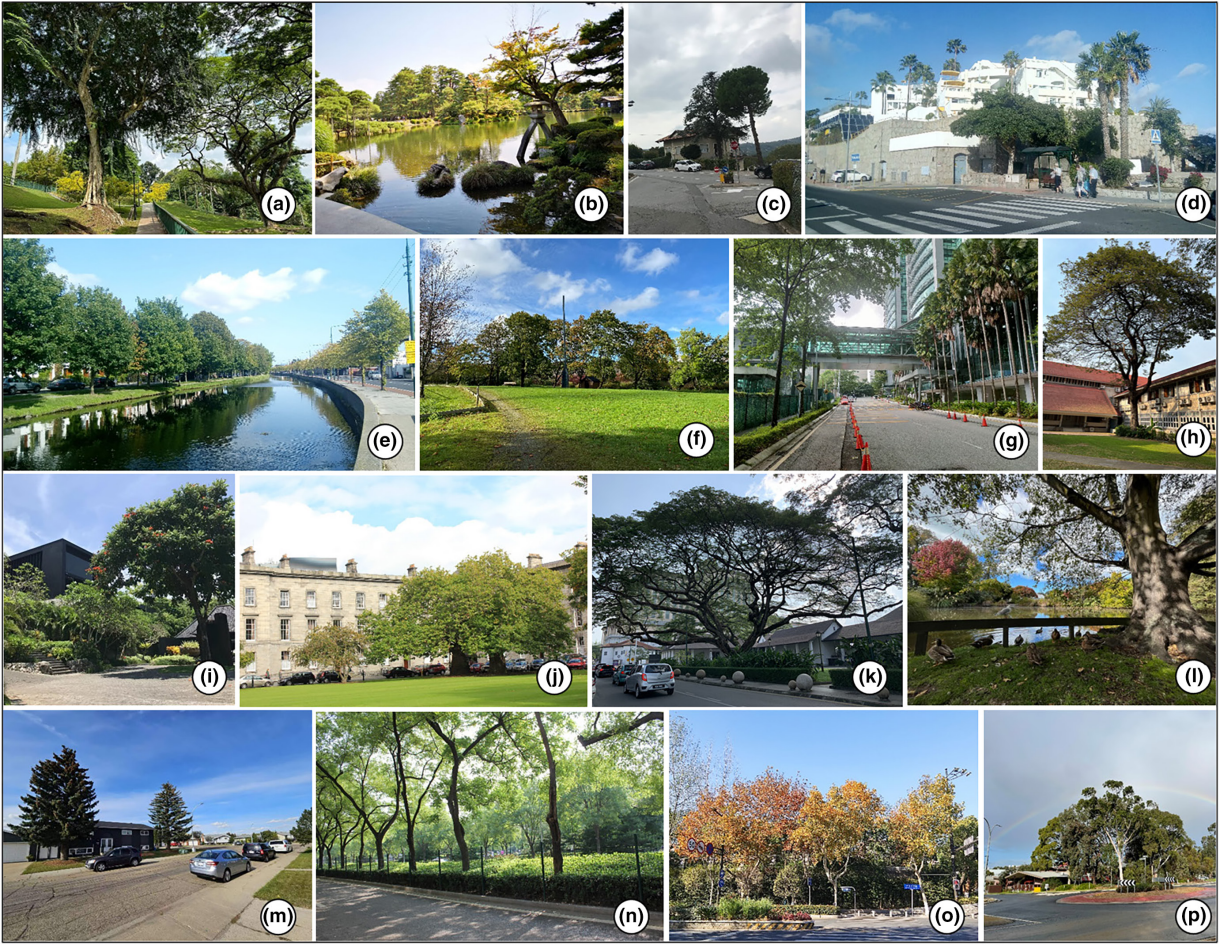
Tree diversity in various countries: (a) Singapore; (b) Ishikawa, Japan; (c) Milan, Italy; (d) Las Palmas de Gran Canaria, Spain; (e, j): Dublin, Ireland; (f) Stavanger, Norway. (g) Kuching, Malaysia; (h) Bangkok, Thailand; (i) Denpasar, Indonesia; (k) Kuala Lumpur, Malaysia; (l) Oamaru, New Zealand; (m) Edmonton, Canada; (n) Beijing, China; (o) Nanjing, China; and (p) Geelong, Australia.

Furthermore, urban trees' cultural, social, and psychological benefits are raised in tandem with diverse species composition that can provide varied blossoms, foliage, forms, seasonal displays, and connection to local ecology (Roy et al., 2012; Salmond et al., 2016). However, urbanization filters and reduces native tree diversity due to habitat degradation, fragmentation and loss, altered environmental conditions, and spread of invasive species (Wang et al., 2020). Protecting and enhancing urban tree diversity through ecologically‐informed planting and stewardship is essential for healthy, resilient, and livable cities. Urban areas can be ecologically engineered to harbor rich tree diversity (Figure 1).

In the 19th and early 20th centuries, many North American cities experienced massive losses of urban tree cover due to major pest and disease outbreaks (Raupp et al., 2006). The high infectivity and mortality were attributed mainly to planting low‐diversity urban forests dominated by a few susceptible species (Conway & Vander Vecht, 2015), such as American elm, American chestnut, and ash. For example, American elms were widely cultivated as resilient street trees in many cities due to tolerance of pollution and road salt (Santamour Jr, 2004). However, the unchecked spread of Dutch elm disease (*Ophiostoma novo‐ulmi*) in the early to mid‐1900s devastated urban elm populations beset by genetic uniformity (Ganley & Bulman, 2016). Elm mortality exceeded 75% in many cities, causing drastic canopy decline, substantial tree removal and replacement costs, and loss of ecosystem services (Copeland et al., 2023). This experience demonstrated the alarming risks of low‐diversity urban tree stocks and the need to switch to disease‐resistant and diverse native species.

Urbanization is accelerating globally, with over 50% of the world's population now living in cities (United Nations, 2019). This rapid expansion and densification of urban areas have exerted increasing pressure on urban forests and tree diversity. Urban development leads to direct and indirect habitat decline due to building and road construction, gray infrastructure projects, and general sprawl of built‐up cover at the expense of natural areas (Bhatta, 2010). The remnant habitat patches become isolated to hinder species dispersal and gene flow (Miles et al., 2019). Soil disturbance, pollution, disrupted hydrology, and modified microclimates eliminate many native tree species that often demand more natural conditions (Kowarik, 2023). Climate change exacerbates the stresses through elevated temperatures and more frequent and intense rains, droughts, and storms that may exceed the ecological amplitude of local tree populations (Haase & Hellwig, 2022).

Additionally, urbanization facilitates invasions by exotic tree and pest species that can outcompete natives, become invasive, and suppress the biodiversity and ecosystem functions (Sage, 2020). Mitigating these multiple direct and indirect stresses on native urban trees requires protecting remaining natural areas, improving connectivity through green corridors, restoring native species diversity, managing invasive species, and creating site conditions suitable for a broad and resilient mix of urban‐adapted species (Duinker et al., 2015). Sustaining healthy urban forests under continued urbanization and changing climate presents ongoing and acute challenges.

Urban forest research has expanded substantially in recent decades. It has decidedly extended toward some special themes, including ecosystem services, management practices, and human dimensions, while biodiversity per se has received less direct attention (Davies et al., 2017; O'Sullivan et al., 2017; Salmond et al., 2016). There is a need to synthesize current knowledge by exploring the patterns, drivers, and dynamics of urban tree diversity (Threlfall et al., 2022). Several reviews have broadly examined urban plant diversity trends (Roy et al., 2012). However, few have synthesized findings and perspectives across disciplines to assess the status and future of urban tree diversity. Important knowledge gaps have persisted regarding biotic homogenization, differentiated diversity responses across geographical and socioeconomic contexts, future climate change projections, and ecosystem resilience implications (Kattel et al., 2013). Targeted synthesis of current interdisciplinary insights on urban tree diversity will help inform resilient urban forest planning and identify critical areas for further research.

However, distilling the extensive literature on urban tree diversity into clear insights and future directions remains challenging without a robust and systematic quantitative analysis. Traditional narrative reviews relying solely on limited publications may introduce personal biases to skew the results (Gough et al., 2017). In contrast, bibliometric analysis enlists mathematical models and statistical techniques to enable more objective assessments of research fields using comprehensive datasets (Nikseresht et al., 2022). The data‐driven approach grounded in quantification elucidates the knowledge structure, revealing research hotspots and emerging directions by delineating the intricate networks between publications, authors, institutions, and concepts (Li et al., 2022).

Applied to the interdisciplinary domain of urban tree diversity, bibliometric techniques can uncover the active topics, influential authors and affiliations, patterns of international collaboration, and linkages with other disciplines from a large publication dataset. Mapping the knowledge topology helps pinpoint critical gaps to steer future studies. The bibliometric method can extract otherwise obscured patterns and trends and unlock embedded scientific visions by aggregating and filtering the substantial publication corpora. With foundations in mathematics, statistics, and computer science, it provides data‐supported insights to benchmark the pulse of research fields (Chen et al., 2023). With intensifying urban sustainability challenges globally, applying bibliometric analysis to synthesize the extensive urban tree diversity literature can crystallize current understanding and illuminate unresolved questions to drive research. The findings can inform setting strategic priorities for researchers, policymakers, and practitioners to scale up green infrastructure worldwide.

This study employed bibliometric analysis to examine comprehensively and systematically the urban tree diversity research domain. The detailed quantitative approach has several aims: (1) evaluate outputs and citation profiles to gauge impacts; (2) identify influential publications through literature co‐citation network analysis; (3) determine key contributing authors, institutions, countries, and journals; (4) elucidate collaboration patterns and their evolution; and (5) delineate hot topics and research frontiers over the study period. This bibliometric study sought to uncover publication and authorship patterns and trends vis‐a‐vis emerging research priorities by quantifying research outputs, mapping citation networks, and delineating collaboration linkages. The goal is to assess objectively the urban tree diversity literature to highlight impactful works, leading contributors, productive collaborations, and critical knowledge gaps to inform future research directions. The findings could synthesize current understanding and provide strategic insights to suggest or steer future studies of urban tree diversity.

## 2 MATERIALS AND METHODS

**FIGURE 2:**
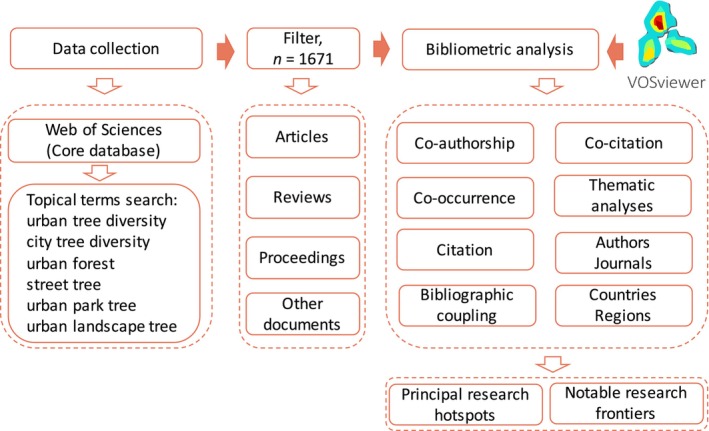
A flowchart depicting the bibliographic search and screening procedures used in this systematic literature review.

The data source for this literature review consisted of publications on urban tree diversity indexed in the Web of Science Core Collection database from 2000 to 2022. The search strategy employed topical terms: “urban tree diversity”, “city tree diversity”, “urban forest”, “street tree”, “urban park tree”, and “urban landscape tree” to capture relevant articles, reviews, proceedings, and other documents. The retrieved records contained comprehensive bibliographic information, including entire paper texts, publication year, journal name, paper title, authors, affiliations, keywords, abstracts, and citation counts. The harvested metadata were exported in CSV format for bibliometric analysis. The July 09, 2023 search retrieved 1671 publications covering the 23‐year study period. Quantitative analysis was conducted by the VOSviewer software (version 1.6.19) to assess the co‐authorship, co‐occurrence, citation, bibliographic coupling, co‐citation, and thematic analyses (van Eck & Waltman, 2010). The comprehensive dataset enables an objective, data‐driven assessment of knowledge patterns and trends in the research field (Figure 2).

VOSviewer enables network mapping and analysis of citations, bibliographic couplings, co‐citations, co‐authorships, co‐occurrences, and research themes (Laengle et al., 2018). Its knowledge graph outputs, in the form of node‐link network maps, vividly depict collaboration links, research hotspots, and knowledge clusters. The text files containing the metadata of retrieved publications were loaded into VOSviewer. Co‐authorship networks were generated to express connections between contributing authors, institutions, and countries. Node size represented the number of publications, while the inter‐node distance indicated collaboration strength. Co‐occurrence networks mapped connections between frequently occurring keywords, with node size portraying the occurrence frequency and distance denoting the co‐occurrence strength (Li et al., 2022).

Additionally, publication dates, contributing journals, author affiliations, and other metadata were analyzed using Microsoft Excel 2019 to generate temporal charts and quantify distribution patterns. Combining VOSviewer and Excel techniques allowed an integrated bibliometric analysis and visualization. This approach enabled an objective examination of research trends, productive contributors, collaboration links, conceptual associations, and knowledge clusters in the urban tree diversity literature over the past two decades.

## 3 RESULTS AND DISCUSSION

### 3.1 Temporal distribution of publications

**FIGURE 3:**
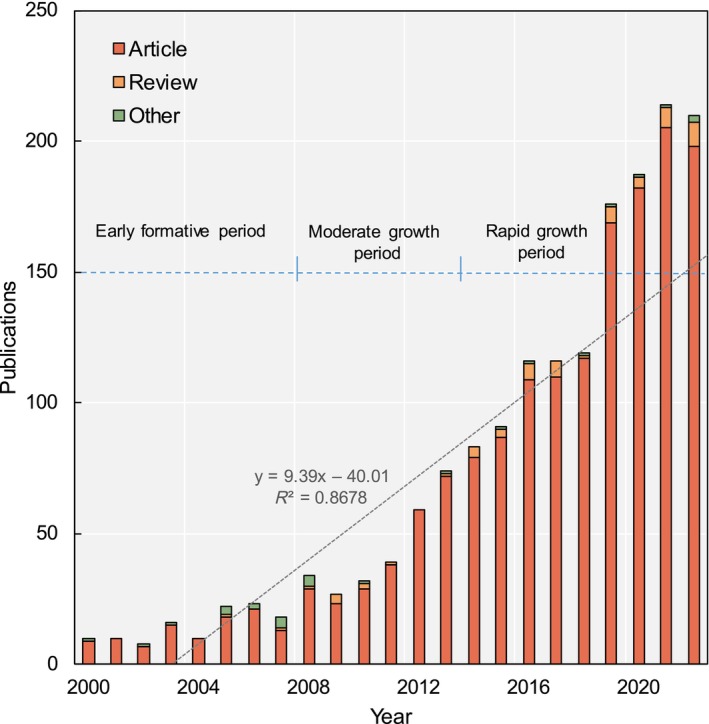
The significant rising trend of publications is divided into three types on urban tree diversity from 2000 to 2022. The linear regression trend line indicates the average rate of change in the study period. The “Article” type refers to journal papers reporting original research findings.

From 2000 to 2022, research outputs on urban tree diversity grew considerably, increasing by 20‐fold from just 9 in 2000 to 207 in 2022 (Figure 3). Some 96% of the publications were journal articles reporting original empirical findings. In the early 2000–2005 period, articles were the only publication type, with no reviews or other categories. This rather monolithic makeup reflects the nascent state of the field, focused on the initial analysis of tree diversity in various cities through data collection and field surveys. The first review appeared in 2005 as the literature began to accumulate. Reviews gradually became more common over time, constituting 3.5% of the outputs by 2022, with 58 reviews published over 23 years. These synthesized insights signaled the field's progression from isolated studies to a more connected and consolidated knowledge body. Other publication types, like reports, first emerged in 2000 but remained scarce and never exceeded 1% in any given year.

The publication trends expressed three distinct developmental phases (Figure 3). The formative 2000–2007 period had a low output of <15 papers per year, all basic diversity descriptions. Moderate growth occurred in 2008–2013, exceeding 20 publications per year by 2008 and reaching 74 by 2013, and reviews and reports began to appear sporadically. Notable expansion happened after 2013, with annual output more than doubled from 83 in 2014 to 213 in 2021. Reviews also became more frequent (Morgenroth et al., 2016), constituting up to 4% of publications by 2021, although articles remained dominate at 95% of the papers. This phase indicated urban tree diversity emerging as a major research focus, with original studies on threats, management strategies, social perspectives, and projections (Roebuck et al., 2022) and reviews synthesizing the burgeoning knowledge base (Nitoslawski et al., 2016).

Overall, the literature has prioritized empirical studies, providing essential data on patterns, drivers, and implications of urban forest diversity. Meanwhile, reviews have gradually increased, offering topical syntheses to complement and synergize insights from hundreds of articles. This ever‐growing publication corpus reflects rising scientific and policy interest in understanding and protecting urban biodiversity in the face of pressing urban challenges.

### 3.2 Notable bibliometric features

#### 3.2.1 Principal research fields

Over the past two decades, research on urban tree diversity has increased across various research fields, with domination by ecology, environmental science, and urban studies (Table 1). In the formative 2000–2007 period, when publication output was limited, ecology accounted for 28% of papers, environmental science 14%, and urban studies 7%. This early phase focused on foundational biodiversity surveys and environmental assessments in city landscapes (Jim & Liu, 2001; Sæbø et al., 2005). The moderate growth in output in 2008–2013 witnessed the continued dominance of ecology with 22% of papers. In comparison, urban studies rose to 12%, and environmental science dropped to 8%, highlighting an increasing emphasis on human factors, planning, and sustainability (Jim, 2013; Roy et al., 2012; Young, 2011). The fast‐expanding post‐2013 period aligned with the emergence of urban tree diversity as a major research priority. Total outputs increased nearly five‐fold from 2008–2013 to 2014–2022. Ecology retained its top position with 17% of papers, followed by environmental science at 16% and urban studies at 13%. However, plant science, biodiversity conservation, forestry, and geography experienced incipient growth in this period, reflecting a broadened concern about threatened urban ecosystems (Kowarik et al., 2020; Livesley et al., 2016).

**TABLE 1:** The distribution of publications on urban tree diversity by the top 10 research fields from 2000 to 2022.

Research field	2000–2007	2008–2013	2014–2022	Total
Ecology	54	113	424	591
Environmental science	27	44	389	460
Urban studies	13	65	333	411
Forestry	8	50	318	376
Environmental studies	13	54	286	353
Plant science	14	56	271	341
Biodiversity conservation	24	42	234	300
Geography physical	15	39	81	135
Geography	13	29	65	107
Regional & urban planning	13	29	63	105
Total	194	521	2464	3179

Over 23 years, ecology led with 19% of the 3179 publications, environmental science and urban studies were tied for the second rank with 14%, and forestry ranked fourth with 12%. The prominence of urban‐focused fields reflected the rising concern about sustainable and livable cities, while the prominence of ecology and conservation echoed biodiversity threats coming to the forefront. As the literature swelled, the scope expanded across disciplines, signaling urban forests' progressively eclectic coverage of environmental, social, economic, and political domains. However, ecology has remained at the core, providing critical biodiversity data and interpretations fundamental to different aspects of scholarship and policy on this complex topic (Locosselli & Buckeridge, 2023).

#### 3.2.2 Main research contributions by country and region

The top 10 countries collectively accounted for over 80% of all outputs, indicating the concentration of research capacity in developed Western nations and emerging economies like China and Brazil. The USA led global scholarship on urban tree diversity, accounting for 22.92% of publications from 2000 to 2022 (Table 2). With 383 articles and over 10,000 citations, US researchers made fundamental contributions across biodiversity assessments, social analyses, and policy guidance (Nitoslawski et al., 2016). China ranked second with 15.08% of papers, reflecting rapid urbanization pressures on its urban forest ecosystems (Jim, 2013). Brazil, Australia, Canada, Germany, the UK, Italy, France, and Poland joined the top 10 producing nations, each contributing 4%–7% of the literature. Notably, the contributions of Australia and Canada highlighted urban forest priorities for these highly urbanized countries (Davies et al., 2017; Hartigan et al., 2021). Though the USA led in volume and influence, urban forest scholarship has spanned several continents, with some nations making disproportionate contributions relative to the global share of urban forests.

**TABLE 2:** The top 10 countries reckoned by the number of publications on urban tree diversity from 2000 to 2022.

Country	Publication	Percent	Total citation	Mean citation	*h*‐index^a^
The USA	383	22.92	10,722	27.99	53
China	252	15.08	4239	16.82	33
Brazil	129	7.72	1505	11.67	21
Australia	110	6.58	3554	32.31	32
Canada	110	6.58	3069	27.90	32
Germany	109	6.52	3307	30.34	33
The UK	89	5.33	3569	40.10	31
Italy	82	4.91	2358	28.76	26
France	74	4.43	2503	33.82	30
Poland	63	3.77	1235	19.60	19

^a^ The *h*‐index measures the productivity and citation impact of the publications of a scholar or the research group. It is defined as the maximum number of *h* papers a scholar or research group has published that have been cited at least *h* times (Hirsch, 2005).

**FIGURE 4:**
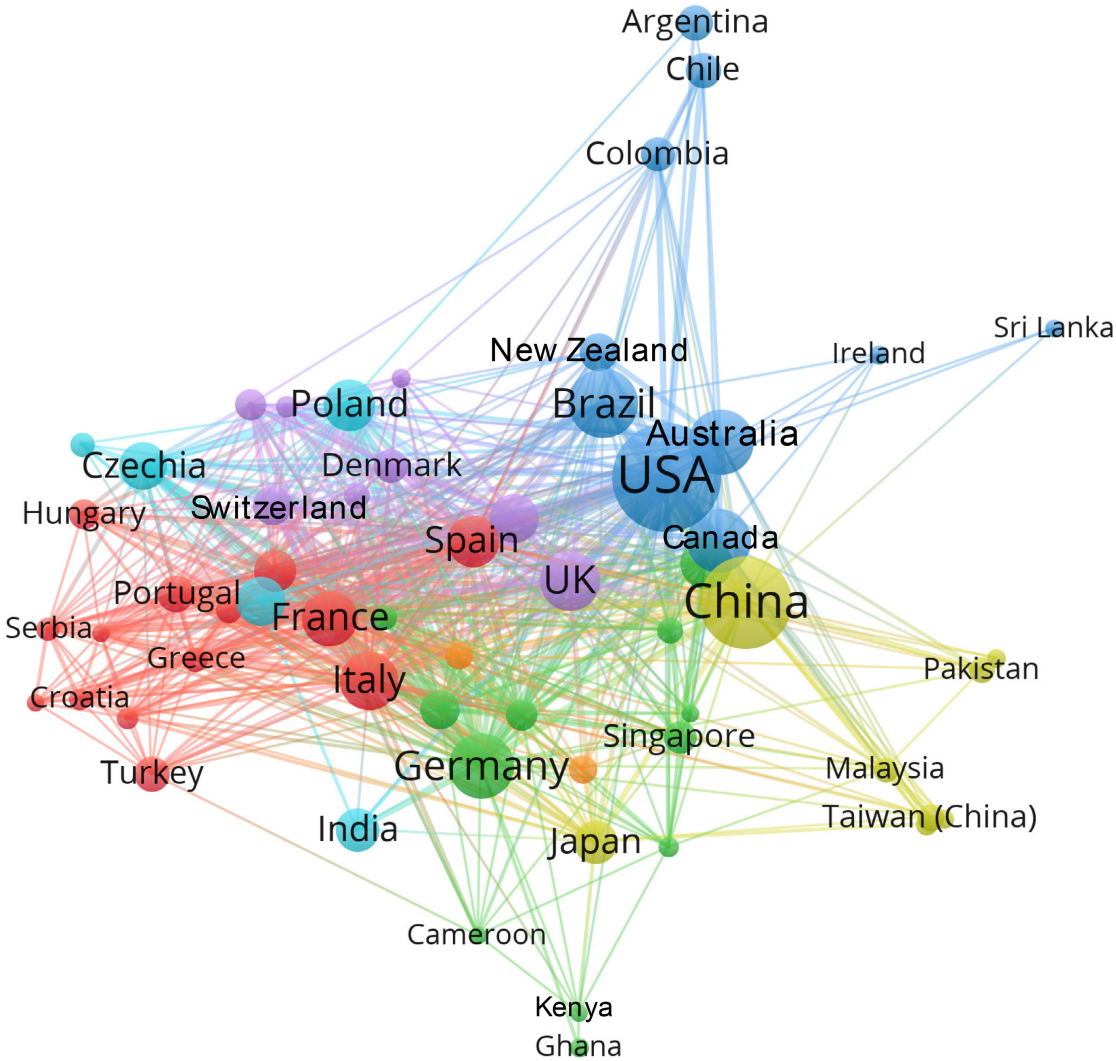
Knowledge graph of the research collaboration network of countries regarding urban tree diversity publications from 2000 to 2022. The font size of a country name is proportional to the number of connections.

Analysis of co‐authorship patterns revealed five distinct clusters of country collaboration in urban tree diversity research from 2000 to 2022 (Figure 4). The red cluster indicated regional research alliances on Mediterranean urban forests. The green cluster reflected cooperation in investigating tropical and developing city landscapes. The blue cluster embodied the core Anglosphere countries. The yellow cluster showed the pattern signified research partnerships across Asia's rapidly growing urban regions. Finally, the Purple cluster demonstrated an expansive reach beyond geographical proximity. Meanwhile, several alliance patterns could be observed. First, proximity facilitated collaboration, as evidenced by Western Europe, Anglosphere, and Asian clusters. Second, shared language and academic systems promoted partnerships like the UK's ties with Europe, Asia, and its former colonies. Third, research capacity appeared crucial—productive entities like the USA, China, and Brazil anchored collaborations with smaller countries. Fourth, shared concerns of climate, biome, and urbanization pressures induced partnerships, such as tropical Africa with Western Europe. Targeted networking initiatives connecting these clusters could enhance idea exchange and capacity building. Overall, more extensive cross‐cluster engagement could further advance the field.

#### 3.2.3 Major research institutions

The Chinese Academy of Sciences was the top contributing institution, with 54 publications, reflecting China's recent rapid expansion in urban ecology research (Table 3). The USDA Forest Service ranked second with 41 papers, underscoring the established strength of US scholarship propped by its universities and extensive government‐agency research. The Swedish University of Agricultural Sciences ranked third with 31 articles, highlighting Sweden's prominence in the field relative to its population size and city count (Morgenroth et al., 2016; Sjoman et al., 2019). The University of Florida and the University of Melbourne rounded out the top 5 with over 25 papers each, demonstrating robust output from these leaders in tropical and temperate urban forestry. The National Autonomous University of Mexico, the University of Hong Kong, the University of British Columbia, the University of the Chinese Academy of Sciences, and Beijing Forestry University completed the list, collectively generating over 200 publications (Jim, 2013; Zambrano et al., 2019). However, the mean citations and h‐indices were often higher for Western institutions versus emerging economy universities. Urban tree diversity scholarship will likely continue to grow in developing countries in the coming decades with the anticipated expansion of research capacity.

**TABLE 3:** The top 10 institutions reckoned by the number of publications on urban tree diversity from 2000 to 2022.

Institution	Publication	Percent	Total citation	Mean citation	*h*‐Index^a^	Country
Chinese Academy of Sciences	54	3.23	1257	23.28	22	China
United States Forest Service	41	2.45	1547	37.73	23	The USA
Swedish University of Agricultural Sciences	31	1.86	1134	36.58	18	Sweden
University of Florida	28	1.68	579	20.68	14	The USA
University of Melbourne	26	1.56	1415	54.42	16	Australia
National Autonomous University of Mexico	25	1.50	424	16.96	12	Mexico
University of Hong Kong	25	1.50	706	28.24	17	China
University of British Columbia	24	1.44	793	33.04	9	Canada
University of the Chinese Academy of Sciences	22	1.32	320	14.55	12	China
Beijing Forestry University	21	1.26	400	19.05	11	China

^a^ Refer to Table 2 for the meaning of the *h*‐index.

**FIGURE 5:**
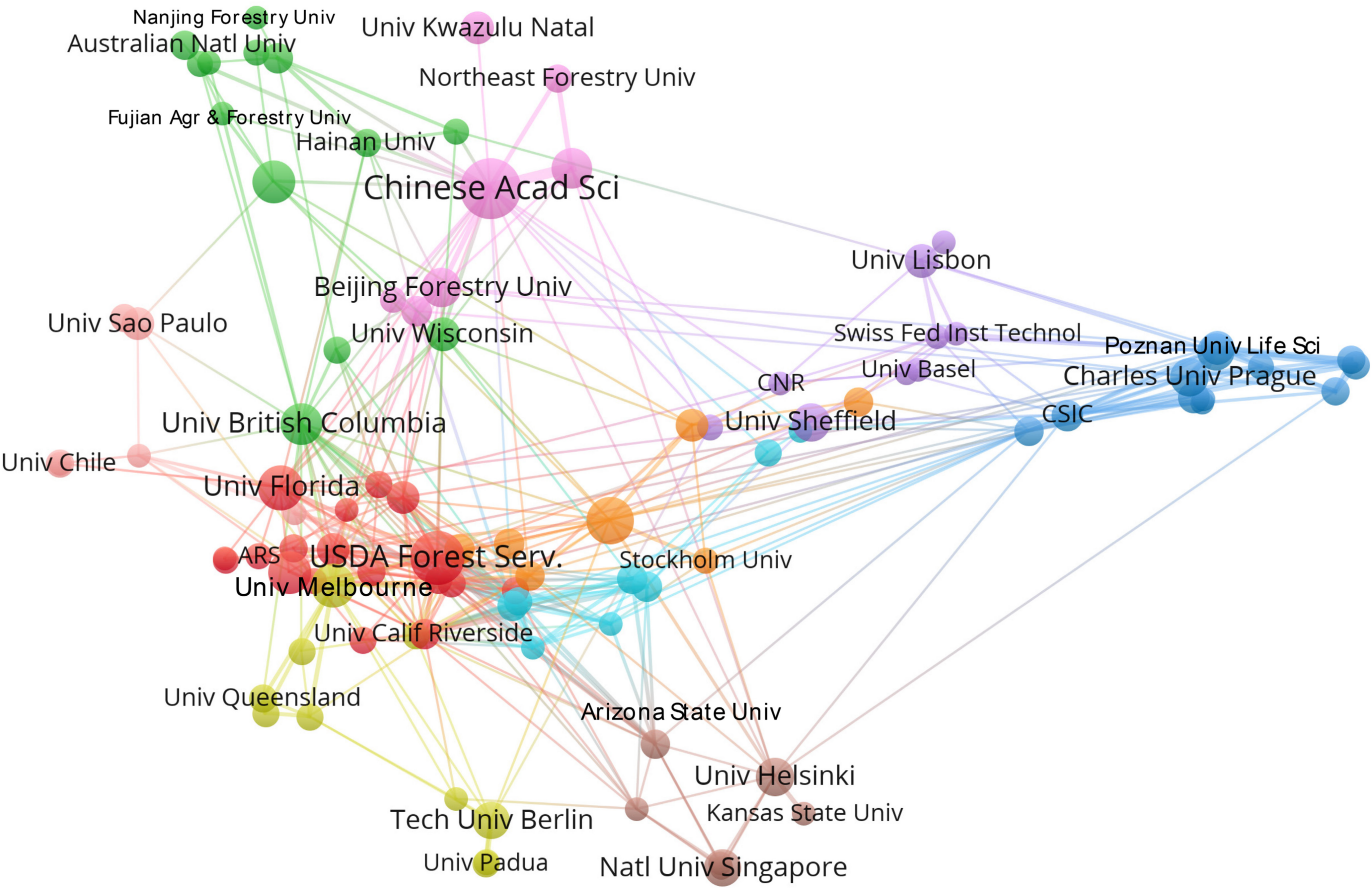
Knowledge graph of the institutional cooperation network involving more than seven publications on urban tree diversity from 2000 to 2022. The font size of an institution name is proportional to the number of connections. ARS, CNR and CSIC denote the United States Department of Agriculture Agricultural Research Service, Consiglio Nazionale delle Ricerche, and Consejo Superior de Investigaciones Cientificas, respectively.

Analysis of co‐authorship networks disclosed five distinct collaborative clusters among institutions with not less than seven joint publications on urban tree diversity from 2000 to 2022 (Figure 5). The red cluster centered on partnerships between the US institutions, pointing to robust intra‐national teamwork. The green cluster linked the University of British Columbia with universities across the Pacific in Hong Kong and mainland China, reflecting pan‐Pacific international cooperation. The purple cluster contained European institutions suggesting regional alliances. The yellow cluster joined highlighted long‐range transcontinental collaboration. Finally, the pink cluster showed extensive co‐publishing among major Chinese institutions.

#### 3.2.4 Key journals by citations

*Urban Forestry & Urban Greening* published the most articles on urban tree diversity, accounting for 11.43% of the total (Table 4). Elsevier publishes it in Germany with high impact metrics, including total citations, impact factor, and journal citation indicator. *Urban Ecosystems* (Springer, New York), *Landscape and Urban Planning* (Elsevier, Amsterdam, the Netherlands), and *Forests* (MDPI, Basel, Switzerland) also published over 60 articles each on this topic. Most journals focused specifically on urban ecology, forestry, and planning, highlighting the multidisciplinary nature of urban tree diversity research. Although the USA produced substantial research outputs on urban tree diversity, the major relevant journals are based in European countries, like the Netherlands and Switzerland.

**TABLE 4:** The top journals reckoned by the number of publications on urban tree diversity from 2000 to 2022.

Journal	Publication	Percent	Publisher	Country	Total citation	IF^a^	JCI^b^
*Urban Forestry & Urban Greening*	191	11.43	Elsevier	The Netherlands	3993	6.4	1.72
*Urban Ecosystems*	115	6.88	Springer	The Netherlands	2089	2.9	0.69
*Landscape and Urban Planning*	103	6.16	Elsevier	The Netherlands	4949	9.1	2.26
*Forests*	63	3.77	MDPI	Switzerland	550	2.9	1.06
*Sustainability*	40	2.39	MDPI	Switzerland	383	3.9	0.68
*Science of the Total Environment*	37	2.21	Elsevier	The Netherlands	1000	9.8	1.67
*Plos One*	30	1.80	Public Library of Science	The USA	714	3.7	0.91
*Ecological Indicators*	25	1.50	Elsevier	The Netherlands	542	6.9	1.49
*Biodiversity and Conservation*	25	1.50	Springer	The Netherlands	1192	3.4	0.75
*Frontiers in Ecology and Evolution*	19	1.14	Frontiers Media	Switzerland	385	3.0	0.74
*Forest Ecology and Management*	19	1.14	Elsevier	Netherlands	674	3.7	1.46
*Biological Conservation*	19	1.14	Elsevier	The UK	1188	5.0	1.42

^a^ A journal's impact factor (IF) is calculated by dividing the number of citations to articles in the previous 2 years by the number of articles published in the same 2 years. The IF data were extracted from the WoS in June 2023.

^b^ The Journal Citation Indicator (JCI) measures the average number of citations received in a given year by the papers published in a journal in the previous 2 years. The worldwide average JCI is set at 1.00. A value above and below 1.00 denotes above or below the world average citation impact, respectively. The JCI data were extracted from the WoS in June 2023.

Seven of the top 12 journals have a JCI (Journal Citation Indicator) above 1.00 regarding citation impacts, indicating above‐world‐average citation impacts. *Landscape and Urban Planning* ranks first with a JCI of 2.26, followed by *Urban Forestry & Urban Greening* with 1.72. These two key journals also score the highest citations. These journals have been pivotal in disseminating scholarly knowledge on urban tree diversity over the past two decades. Their leadership position and preferred venues for urban forest research outputs will likely linger.

#### 3.2.5 Key authors

C.Y. Jim from the Education University of Hong Kong (previously affiliated with the University of Hong Kong) published the highest number of articles (27) on urban tree diversity as the leading author (Table 5). Other prolific authors include MacGregor‐Fors from the University of Helsinki (13 articles), Sjöman from the Gothenburg Botanical Garden (12 articles), and Duinker from the Dalhousie University (11 articles). The top authors came from diverse research institutions worldwide, including North America, Europe, China, and Australia. Most were affiliated with universities that include studies on environmental science, forestry, horticulture, and urban planning, such as the University of Melbourne and the Czech University of Life Sciences. The top publishing authors generally have h‐indexes ranging from 8 to 14, indicating their research influence. The articles from these productive authors have significantly contributed to the literature on urban tree diversity. More cross‐country and cross‐disciplinary collaborations could further enhance the productivity and impact of global urban tree diversity research.

**TABLE 5:** The top authors with more than 10 publications on urban tree diversity from 2000 to 2022.

Author	Publication	Institution	Country	*h*‐Index^a^
Jim, C Y	27	Education University of Hong Kong and University of Hong Kong	China	14
MacGregor‐Fors, Ian	13	University of Helsinki	Finland	13
Sjöman, Henrik	12	Gothenburg Botanical Garden	Sweden	10
Duinker, Peter N.	11	Dalhousie University	Canada	8
Avolio, Meghan L	11	Johns Hopkins University	USA	9
Tryjanowski, Piotr	11	Poznan University of Life Sciences	Poland	8
Escobedo, Francisco J	10	USDA Forest Service	USA	8
Morelli, Federico	10	Czech University of Life Sciences	Czechia	9
Ordóñez, Camilo	10	University of Melbourne	Australia	8
Williams, Nicholas S G	10	University of Melbourne	Australia	9
Kendal, Dave	10	University of Tasmania	Australia	8

^a^ The *h*‐index here specifically refers to an author's influence only in the field of urban tree diversity rather than the influence of all publications of an author.

**FIGURE 6:**
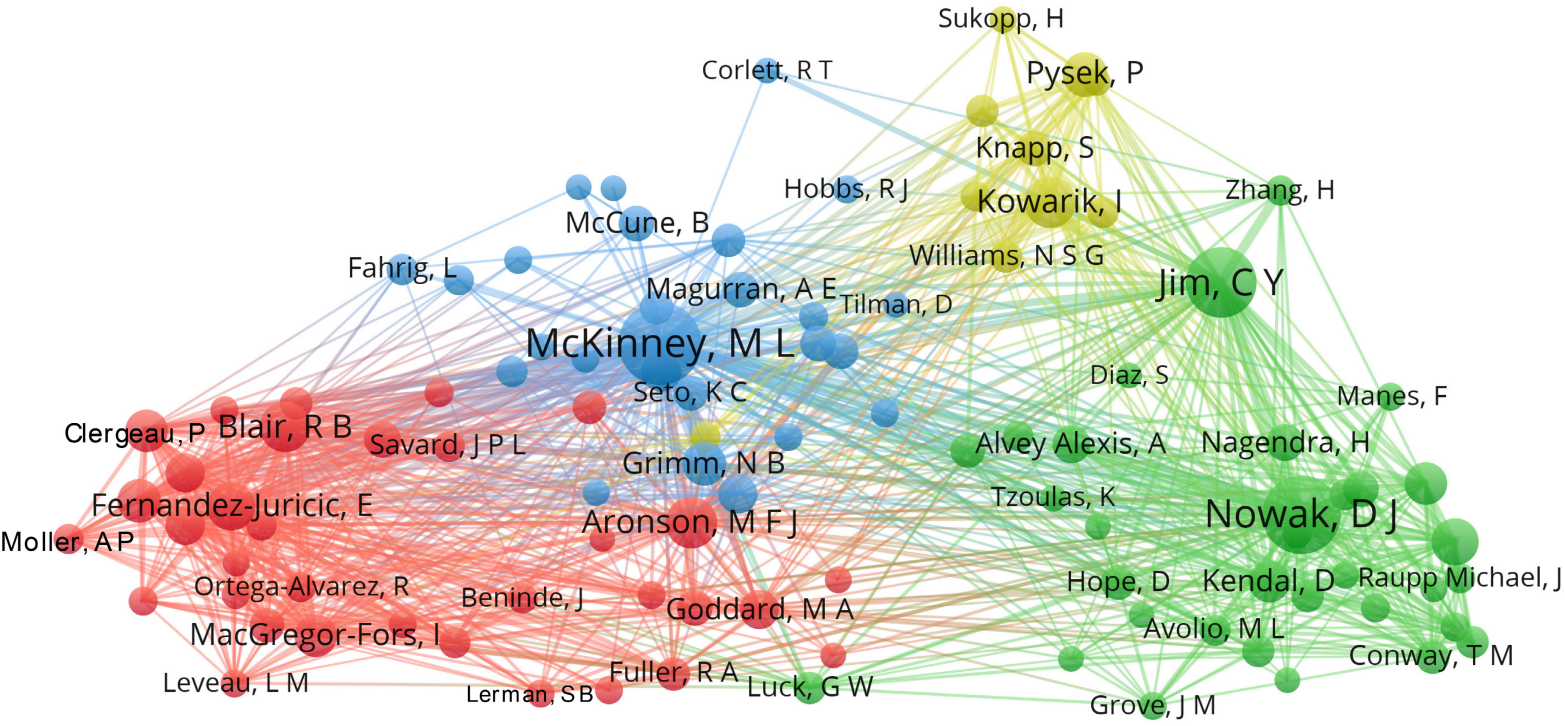
Knowledge graph of authors' co‐citation regarding urban tree diversity publications from 2000 to 2022. The font size of an author name is proportional to the number of connections.

The co‐citation analysis identified four major clusters of influential authors in urban tree diversity research from 2000 to 2022 (Figure 6). The red cluster contained authors focusing on urban ecology and conservation, like Anderson, Clergeau, Savard, and Moller (Clergeau et al., 2006; Rega‐Brodsky et al., 2022). The green cluster represented leading researchers on urban forestry and green infrastructure, including Jim, Nowak, McPherson, Kendal, and Escobedo (Escobedo et al., 2019; Jim et al., 2018). The blue cluster covered seminal community and landscape ecology authors like McDonnell, Pickett, Magurran, and Hobbs (McDonnell & Hahs, 2008; Zhou et al., 2017). Finally, the yellow cluster comprised key scientists studying biological invasions and plant diversity like Kowarik, Pysek, Knapp, and La Sorte (Kowarik et al., 2020; Pyšek et al., 2004). The analysis reflected the multidisciplinary nature of urban tree diversity research spanning urban ecology, forestry, invasion biology, community ecology, and landscape ecology. While some prominent cross‐citations existed between clusters, the four groups also denoted distinct intellectual silos. More interdisciplinary collaboration and co‐citation linkages between the clusters could benefit future urban tree diversity research. The co‐citation patterns illustrated the field's evolution over 20 years through its pioneering members and their scholarly connections.

#### 3.2.6 Most cited publications

The most cited publication was about urban domestic gardens (Gaston et al., 2005), highlighting the biodiversity value of neglected private urban green spaces and catalyzed mapping studies (Table 6). The second most cited publication was a pioneering modeling study on the environmental effects of urban change and the dynamics of greenspace (Pauleit et al., 2005). Therefore, the most highly cited works over the past 20 years displayed several key trends shaping the trajectory of urban tree diversity research.

**TABLE 6:** The top 10 most cited publications on urban tree diversity from 2000 to 2022.

First author	Year	Journal	Country/First institution	Topic	Citation
Gaston, K J	2005	*Biodiversity and Conservation*	UK/University of Sheffield	Biodiversity & Conservation	384
Pauleit, S	2005	*Landscape and Urban Planning*	UK/University of Manchester	Regional & Urban Planning	358
McDonnell, M J	2008	*Landscape Ecology*	Australia/Royal Botanic Gardens Melbourne	Ecology	336
Jumpponen, A	2009	*New Phytologist*	USA/Kansas State University	Plant Science	316
Chytry, M	2008	*Ecology*	Czechia/Masaryk University	Ecology	311
Melles, S	2003	*Conservation Ecology*	Canada/University of Toronto	Ecology	293
Gregg, J W	2003	*Nature*	USA/Cornell University	Science & Technology	292
Whitford, V	2001	*Landscape and Urban Planning*	UK/University of Manchester	Environmental Science & Ecology	277
Mathieu, R	2007	*Landscape and Urban Planning*	New Zealand/University of Otago	Geography	270
Sandstrom, U G	2006	*Landscape and Urban Planning*	Sweden/Örebro University	Ecology	268

First, early studies established baseline documentation and mapping of tree diversity patterns across urban landscapes, providing critical knowledge and revealing immense data gaps (Gregg et al., 2003). This development catalyzed the application of new technologies, including remote sensing, big data analytics, and molecular sequencing, to investigate diversity at novel scales (Mathieu et al., 2007). Second, pioneering modeling approaches demonstrated their potential under environmental change and urbanization, highlighting the need for predictive tools to guide proactive planning (Pauleit et al., 2005). Consequently, simulation studies began flourishing, including investigating socioeconomic drivers to ecological outcomes.

Third, gradients and landscape frameworks emerged as key conceptual lenses to analyze how processes across spatial scales influence biodiversity (McDonnell & Hahs, 2008). This new trend pump‐prime the integration of landscape ecology and macroecology perspectives into the field. Fourth, early works highlighted the complexity of diversity by unraveling differences between native/exotic, trait/taxonomic, phylogenetic, and genetic diversity (Sandström et al., 2006). This realization led to studies disentangling the multiple facets of diversity. Finally, seminal research on ecosystem service quantification helped strengthen the applied aspects of biodiversity studies. In summary, these innovative works inspired major theoretical and methodological advances. They enabled the multifaceted, multiscale, socio‐ecological systems approach that defines contemporary urban tree diversity research.

### 3.3 Principal research hotspots

**FIGURE 7:**
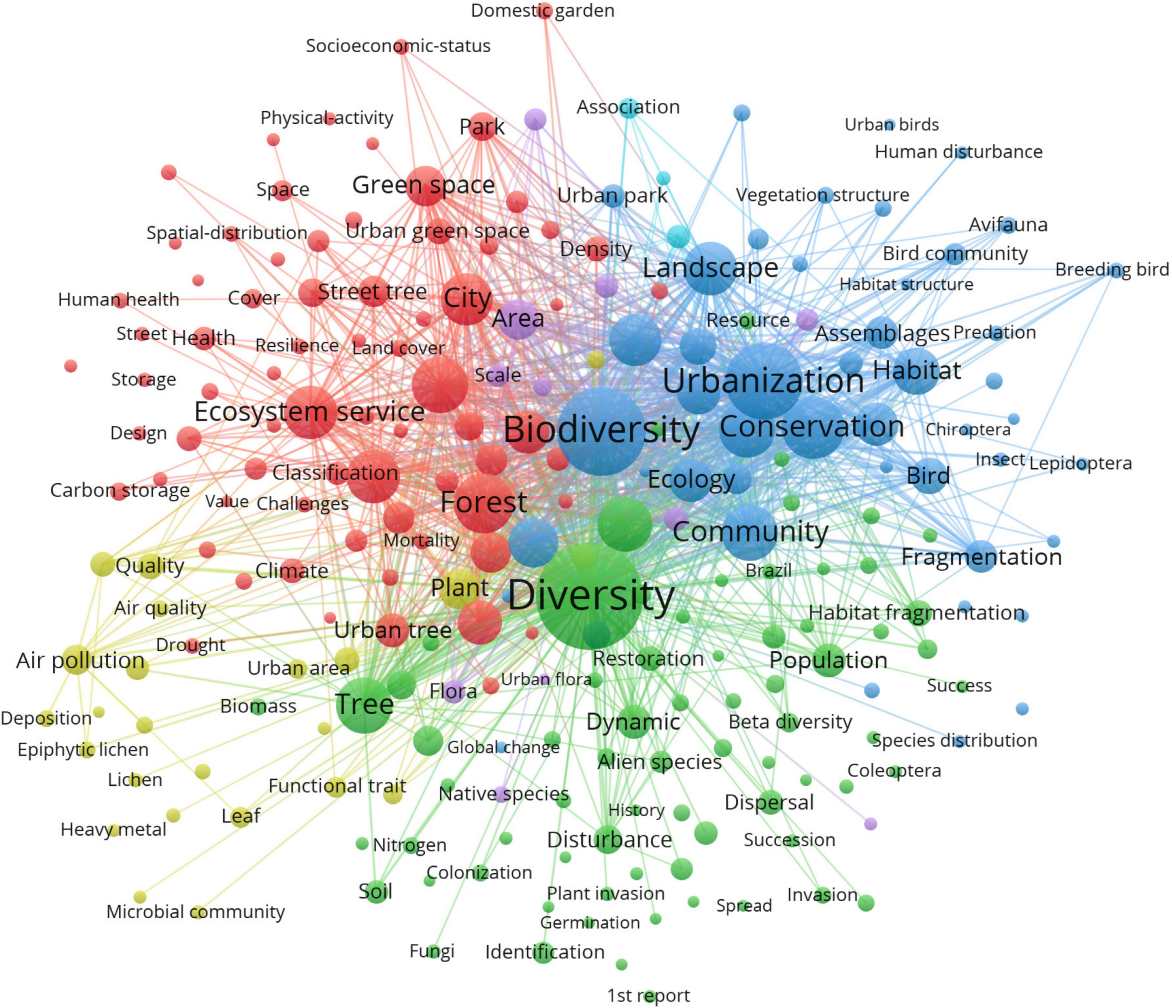
Knowledge graph of keyword co‐occurrence and clustering regarding urban tree diversity publications from 2000 to 2022. The font size of a keyword is proportional to the number of connections.

From the knowledge graph presented in Figure 7, four principal groups of research hotspots could be identified:

(1) Urban tree diversity research coalesced around several interconnected hotspots. They included forest structure and composition, ecosystem services, biodiversity patterns, environmental drivers, and sustainable management (Figure 7, red cluster). Studies of urban forest vegetation quantified tree communities across cities to catalog species diversity while revealing differences based on climate, disturbance history, socioeconomics, and biogeography (Esperon‐Rodriguez et al., 2020; Jim, 2013; Salmond et al., 2016). Assessing ecosystem services became a major research priority, with studies valuing the contributions of urban forests to air pollution removal, carbon sequestration, stormwater mitigation, and physical and mental health (Roebuck et al., 2022; Roy et al., 2012; Salmond et al., 2016). Biodiversity research examined how urbanization impacts species diversity and community tree assemblages, understory plants, birds, insects, and soil microbiota (Livesley et al., 2016; Sandström et al., 2006). Environmental drivers such as land use change, climate change, invasive species, pollution, and urban heat islands were investigated for their effects on urban forest diversity (Bourne & Conway, 2014; Morabito et al., 2021; Nitoslawski et al., 2016; Threlfall et al., 2022).

Sustainable urban forest management demonstrated a growing focus, with efforts to balance competing priorities of vegetation density, species diversity, public safety, esthetics, and resilience (Jim, 2013; Kowarik et al., 2020). To inform management, studies modeled future urbanization scenarios, landscape connectivity, species distributions, and implications of climate change (Roy et al., 2012; Stas et al., 2020). Research on the human dimension evaluated public perceptions, behaviors, and governance related to urban trees. Synthesizing findings across disciplines, scales, and methods enabled a holistic understanding of linkages between urban tree diversity and human well‐being (Morgenroth et al., 2016; Zambrano et al., 2019). With urbanization accelerating worldwide, transdisciplinary collaborations, comparative studies across cities, and linking science with policy and practice would be critical to translating urban tree diversity research into effective management solutions for resilient, sustainable, and biodiverse cities globally.

(2) Understanding patterns, dynamics, and drivers of tree diversity presented a major research priority for urban ecosystems worldwide (Figure 7, green cluster). Studies analyzed alpha and beta diversities to quantify variations in community composition between sites and regions (Bourne & Conway, 2014; Ricotta et al., 2012). Biodiversity mapping projects documented population distributions, revealing citywide gradients and hotspots (Esperon‐Rodriguez et al., 2020; McDonnell & Hahs, 2008; Wang et al., 2020). Examining temporal dynamics provided insights into colonization, extinction, and turnover as impacted by urbanization. Demographic research tracked tree growth, recruitment, and mortality, with findings often indicating reduced regeneration (Haase & Hellwig, 2022; Raupp et al., 2006; Smith et al., 2019). Investigating population dynamics involved mapping size and age structures to identify sustainable cycles versus impending loss. Fragmentation was studied as a driver of diversity, with habitat patches supporting distinct communities separated by barriers (Mavimbela et al., 2018). Connectivity research assessed dispersal limitations and landscape connectivity to guide restoration (Bierwagen, 2007).

Studies also considered trait diversity involving tolerances, growth rates, and functions (Conway & Vander Vecht, 2015; Haase & Hellwig, 2022; Roebuck et al., 2022). Disturbances altered competitive hierarchies between native and invasive trees, impacting diversity (Johnson & Handel, 2016; Wang et al., 2020). Interactions between urbanization drivers such as heat islands and impervious surface and tree diversity were examined (Morabito et al., 2021). Changing environmental conditions due to land use and climate change and implications for biodiversity were active research frontiers (Bourne & Conway, 2014; Burley et al., 2019). Findings were applied to assessing ecosystem services, modeling vegetation shifts, predicting future distributions, and managing diversity. Comparative urban diversity studies along latitudinal and environmental gradients provided the spatial context to understand macro‐scale patterns (Esperon‐Rodriguez et al., 2020; Wang et al., 2020). Integrating diversity patterns, processes, and projections enabled deeper ecological understanding and informed global sustainable urban forest planning and management.

(3) Biodiversity conservation in urban ecosystems demonstrated an advancing frontier, with studies quantifying tree diversity through species inventories and richness assessments across metropolitan landscapes (Figure 7, blue cluster). The research characterized urbanization impacts on community composition, often finding reduced native tree cover and shifts toward non‐native and ornamental species (Gregg et al., 2003; Ruas et al., 2022). Land use analyses associated higher diversity with parks, gardens, and riparian areas, contrasting with lower diversity in heavily paved areas (Bourne & Conway, 2014; Nitoslawski et al., 2016). Examining urbanization as a gradient facilitated comparisons of tree communities along rural‐suburban‐urban transects and between cities differing in density and sprawl (Wang et al., 2020). Landscape ecology approaches assessed habitat patches, fragmentation, and connectivity to understand compositional patterns (Norton et al., 2016). Remote sensing, field surveys, and citizen science datasets were combined to map and monitor communities at broader scales (Ozkan et al., 2016; Robinson et al., 2020). Network analysis quantified biodiversity distribution across green space networks. Comparative studies along latitudinal, climatic, and biogeographic gradients provided the regional context while revealing global patterns of biotic homogenization and diversity filtering in cities (Aronson et al., 2016).

Experimental studies in gardens and field sites provided a process‐based understanding of diversity drivers (Gaston et al., 2005; Mathieu et al., 2007). Biodiversity projections incorporated urbanization scenarios and environmental changes. Multiple facets were analyzed, from species composition to functional to phylogenetic diversity, to support conservation planning and prioritization (Pyšek et al., 2004; Ricotta et al., 2012; Stas et al., 2020). The findings informed practical measures to increase native species establishment in new and existing habitat patches, restore connectivity through corridors, optimize site and species selection in greening projects, and balance various facets of biodiversity effects on human well‐being. Mainstreaming biodiversity into urban planning and policy indicated an increasing emphasis on improving the urban milieu to benefit nature and people (Carroll et al., 2022). Synthesis of conservation science with governance, social values, and design principles shaped diverse, resilient urban tree communities yielding critical ecosystem services.

(4) Urban trees provided critical ecosystem services, with improving air quality an emerging priority (Figure 7, yellow cluster). The research characterized plant traits and functions related to pollution removal, investigating leaf and canopy properties that could enhance air filtration and absorption (Esperon‐Rodriguez et al., 2020; Grote et al., 2016). Assessments were conducted to quantify pollution removal rates for common tree species, with databases developed to inform selection (Grote et al., 2016). Functional diversity studies involved categorizing trees based on traits to maximize service delivery, such as particulate capture and carbon sequestration. Green space networks with diverse functional traits could increase cumulative services.

Leaf and soil chemical analyses detected bioindicators and monitored pollution impacts on vegetation health (Azzazy, 2020; Molnár et al., 2020). Comparative studies found pollution tolerance thresholds across species (Shahrukh et al., 2023). Experimental exposure trials determined damage symptoms and mechanisms (Haase & Hellwig, 2022). Chemical, morphological, and genetic adaptations to pollutants indicated active research areas (Banerjee et al., 2022). Air pollution effects on growth, reproduction, and mortality rates were quantified. Interactions between pollutants and other stressors like drought and pests were studied (Azzazy, 2020). Investigating environmental justice dimensions involved mapping pollution removal across neighborhoods, with findings often highlighting inequities (Pope et al., 2016). Modeling future air quality and health benefits of urban tree planting initiatives helped prioritize communities (Locosselli & Buckeridge, 2023; Roy et al., 2012).

Assessing cost‐effectiveness demonstrated value compared to engineering solutions (O'Sullivan et al., 2017). Developing low‐maintenance trees suited for harsh urban environments indicated a practical goal for the management (Sæbø et al., 2005; Santamour Jr, 2004). Synthesizing research on pollution mitigation, vegetation sensitivity, and adaptive capacities could guide the selection of hardy, high‐functioning species for air quality improvement. Mainstreaming findings into urban forest planning and policy could ensure environmental quality as an integral greening goal. Multifunctional urban tree diversity could be leveraged through strategic planting and management to enhance public health and livability in changing urban environments (Singh et al., 2020).

### 3.4 Notable research frontiers

The bibliometric analysis provided hints to identify the notable research frontiers:

(1) Understanding and modeling future distributions, traits, and ecosystem services of urban trees under climate change was a critical and increasingly nurtured research frontier (Burley et al., 2019). As climate change could lead to warming temperatures, altered precipitation patterns, and more frequent extreme weather events globally, researchers focused on predicting such impacts on urban forests (Pretzsch et al., 2017). The studies applied climate projection and species distribution models to forecast changes in tree growth, mortality, reproduction, phenology, physiology, and habitat ranges under different emission scenarios (Gregg et al., 2003; Smith et al., 2019). The findings could enable more accurate predictions of future urban vegetation states and ecosystem services provisioning.

Specific research questions involved whether warming minimum temperatures could expand southern tree species range but increase the mortality of northern species adapted to colder climates (Brune, 2016). The resulting phenology changes were examined, including earlier spring budburst, delayed autumn senescence, longer growing seasons, and mismatches with pollinators or herbivores (Esperon‐Rodriguez et al., 2020). Plasticity in drought tolerance traits and acclimation abilities were evaluated to determine resilient versus vulnerable species (Haase & Hellwig, 2022). Population genomic approaches assessed the capacity for adaptation (Housset et al., 2018).

Experiments evaluated precipitation and temperature trends to quantify climate change effects directly (Ettinger et al., 2019). Climate velocity models projected migration rates in response to shifts in isotherms poleward and upward in elevation (Holsinger et al., 2019). The results identified dispersal lags and extinction risks in fragmented urban landscapes. The findings were incorporated into process‐based models projecting growth, mortality, regeneration, and biomass under varying climate scenarios (Kowarik, 2023; O'Sullivan et al., 2017). Outputs were applied to assess future ecosystem services like carbon storage, air pollution removal, and stormwater mitigation (Davies et al., 2017; Salmond et al., 2016). Comparative studies along urban climate gradients provided analogs for future conditions. Such research could support climate‐adaptive planning and management to foster resilient, biodiverse, and functional urban forests.

(2) Research was conducted to quantify the relationships between urban tree diversity, human health, and environmental justice. As cities grappled with issues of inequality, studies queried how tree canopy cover, species composition, and access to nature correlated with health outcomes across neighborhoods (Threlfall et al., 2022). Some researchers employed spatial analysis and statistical models to evaluate how the canopy cover in residential areas could relate to outdoor physical activities, the prevalence of chronic diseases, cardiovascular mortality, mental health, social cohesion, and crime rates (Livesley et al., 2016; Salmond et al., 2016). The ecosystem services of trees in providing shade, cooling, and desirable spaces were quantitatively connected to public health (Elderbrock et al., 2020; Salmond et al., 2016). Comparisons across socioeconomic gradients examined distributional equity in exposure to heat islands and air pollution relative to tree cover (Fan & Sengupta, 2022). Field measurements and satellite imagery were used to map canopy cover, land surface temperatures, and high‐resolution vegetation indices across cities (Mathieu et al., 2007).

Controlled experiments studied how interacting with diverse vegetation versus artificial environments could affect stress, cognition, and emotions (Gidlow et al., 2016). Biodiversity experiments manipulated species richness and assessed benefits to concentration, impulse control, and emotional well‐being (Locosselli & Buckeridge, 2023). The findings expounded on urban nature's positive, direct and indirect impacts on public health (Singh et al., 2020). Environmental justice research focused on tree canopy deficiencies of low‐income and ethnic minority communities (Wolch et al., 2014). Urban heat mapping found many underserved neighborhoods disproportionately hot with less mitigation by green space (Calderón‐Argelich et al., 2021; Fan & Sengupta, 2022).

Community‐based participatory research engaged residents in assessing needs and priorities. Inventory data revealed the patterns and magnitude of inequitable tree maintenance across districts. As expanding canopy cover was linked to health improvement, researchers recommended prioritizing greening initiatives and tree planting in marginalized communities to attain equity and justice (Pope et al., 2016). Citizen science apps empowered residents to map their neighborhoods and advocate for change. The fast‐growing research on urban greening, citizen health, and justice is critical for guiding policies, planning, and interventions to foster greener, cooler, healthier, and more equitable cities.

(3) Sustainable urban forest management strategies were developed to optimize city trees' multiple ecological, social, and economic functions. Urban forests provided diverse services, from air purification to esthetic beauty (Duinker et al., 2015), but maximizing one benefit can compromise others (O'Sullivan et al., 2017). Multicriteria decision analyses and scenario planning tools could balance competing objectives (Bizikova & Krcmar, 2015). Specifically, the research used analytical hierarchy processes to weigh and rank priorities for attributes like biodiversity, carbon sequestration, stormwater mitigation, air cooling, noise reduction, property values, public safety, and cost‐effectiveness (Aronson et al., 2016; Clergeau et al., 2006).

Advanced simulation models were applied to test different planting regimes, maintenance schedules, and climate adaptation plans to quantify trade‐offs between goals like native diversity versus resilience (Pauleit et al., 2005; Pretzsch et al., 2017). Optimization algorithms identified management plans to maximize multivariate benefits within budget constraints (Song et al., 2020). However, pure optimization could lead to monocultures like London plane trees that could maximize services at the expense of reduced biodiversity (Haase & Hellwig, 2022; Sæbø et al., 2005). Scenario planning developed quantitative models representing different management paradigms to facilitate stakeholder deliberation and value‐focused priorities.

It was recommended to integrate adaptive planning cycles to monitor indicators and periodically reassess urban forest structures and functions (Escobedo et al., 2019; Hartigan et al., 2021). Climate change could bring species suitability shifts and societal values changes, demanding flexible yet systemic management (Holsinger et al., 2019; Housset et al., 2018; Ruas et al., 2022). Adding equity considerations like environmental justice and equal access to nature required dynamic reallocation of resources over time and space.

Ultimately, researchers applied decision science, simulation, spatial analysis, and participatory tools to develop integrated, sustainable urban forest plans (Hartigan et al., 2021). Rather than siloed with reactive management over‐optimizing a service at the expense of others, cities could benefit from proactive strategies to maximize multifunctionality, flexibility, and community involvement (Hawthorne et al., 2015). Comprehensive, value‐focused urban forest management could offer the key to equitably and sustainably accessing nature's diverse benefits and improving human health, well‐being, and quality of life.

(4) Studies were conducted to leverage new technologies like remote sensing, drones, mobile apps, and simulation models to enhance research and monitoring over large urban areas. High‐resolution satellite imagery, aerial LiDAR scans, and hyperspectral drones were applied to map tree canopy structure, species composition, and functions at sub‐meter resolution across entire cities (Berland & Lange, 2017). Cloud computing enabled the processing of terabytes of geospatial urban forest data into biodiversity indices, microclimate maps, and environmental models (Le & Bakaeva, 2023; Li et al., 2022). Researchers harnessed crowd‐sourced citizen science through cell phone apps, empowering residents to map, identify, and collect data on neighborhood trees (Hawthorne et al., 2015; Robinson et al., 2020). Municipal tree inventories combined with ground‐level observations could scale up via modeling to citywide mappings of species distributions, diversity patterns, and vulnerabilities (Mathieu et al., 2007).

As urban tree databases grew globally, big data analytics effectively identified socioeconomic, planning policy, and biogeographic drivers of diversity (Carroll et al., 2022; Kendal et al., 2014; Ruas et al., 2022; Sage, 2020). Simulation models manipulated virtual urban forests to predict the impacts of future scenarios like invasive pests, climate change, or planting regimes on biodiversity, risks, and ecosystem services. Digital twins merged real‐time sensor data with simulations to improve model accuracy. Researchers could survey urban forests at unprecedented scope and resolution by combining publicly available satellite imagery with crowd‐sourced citizen science data (Robinson et al., 2020).

Cloud computing made large‐scale urban tree data accessible for grassroots discovery and planetary awareness (Hartling et al., 2021). As digital maps and tools engaged communities in research and stewardship, a vision emerged where every tree in every city is sustainably managed using technologies scaling local monitoring to global knowledge sharing (Berland & Lange, 2017). Advanced technologies created better and more opportunities to understand urban tree diversity, drivers, and services with revolutionary breadth and depth.

Additional research priorities in the urban tree diversity domain can be found beyond the emerging frontiers. For instance, studying propagation, establishment, and trait plasticity can guide climate‐resilience efforts by identifying species and cultivars capable of acclimating or adapting to rapid urban environmental change through assisted migration (Esperon‐Rodriguez et al., 2020; Housset et al., 2018). Examining gene flow and evolutionary dynamics between urban and rural tree populations can help understand urbanization as a driver of divergent natural selection and ecotypic variation (Miles et al., 2019). Developing best practices to maximize diversity and ecosystem services in green infrastructure installations, like green roofs and bioretention cells, is needed to improve multifunctionality (Wooster et al., 2022). Finally, modeling trade‐offs and synergies between tree diversity, density, and services can help inform optimal, balanced planting goals and species selection (Kendal et al., 2014; O'Sullivan et al., 2017). As urban forests expand worldwide, research can aim at enlightening resilience, evolution, eco‐technology performance, and planning for multibenefit systems. Targeted studies in these areas can allow sustainable management to keep pace with global urbanization and expectations.

## 4 CONCLUSION

Urban forests and trees provide critical services and benefits in cities worldwide, from improving the human health and well‐being to mitigating climate change and urbanization impacts. However, as global urbanization accelerates and intensifies, successfully integrating trees into the built environment requires proactive and innovative urban forest management for resilience and sustainability.

Recent research has enlightened the importance of biodiversity and native species in promoting ecosystem functions and services. As urban tree diversity is often low, the findings offer ample evidence to maximize diversity with appropriate native species to enhance productivity, stability, and recovery from stressors like pests, disease, and climate extremes. Beyond diversity, urban tree traits and functional characteristics must align with local environmental conditions and management objectives. Understanding how urban stressors filter species traits and drive community assemblages is crucial for urban greenery planning. Despite the stressful, if not aggressive filters of urban habitats, emerging findings show that cities can harbor substantial tree biodiversity due to high habitat heterogeneity and successful species adaptations. Preserving diversity at different levels requires protecting remnant native vegetation, connecting green space patches, and restoring multilayered biomass structures.

Research frontiers for sustainable urban forest management include predictive modeling, advanced technologies such as remote sensing, and community‐based approaches. Cities can optimize urban forest resilience, services, and biodiversity by linking urban tree ecology with human values, equity, and adaptive policies. Sustaining urban trees necessitates an interdisciplinary systems perspective guiding tree selection, placement, and stewardship to meet the goals of livable and sustainable cities.

## AUTHOR CONTRIBUTIONS

**Chunping Xie:** Data curation (equal); methodology (equal); software (equal); supervision (equal); writing – original draft (equal). **Shuifei Chen:** Data curation (equal); investigation (equal); visualization (equal). **Dawei Liu:** Formal analysis (equal); investigation (equal). **Chi Yung Jim:** Formal analysis (equal); software (equal); validation (equal); writing – review and editing (equal).

## FUNDING INFORMATION

This research was funded by the National Natural Science Foundation of China (grant number: 32360417), the Hainan Provincial Natural Science Foundation of China (grant number: 423MS061), and the Education Department of Hainan Province (project number: Hnky2023ZD‐17).

## CONFLICT OF INTEREST STATEMENT

The authors declare that they have no known competing financial interests or personal relationships that could have appeared to influence the work reported in this paper.

## Data Availability

The Web of Science (WoS) data can be accessed through the official website: https://www.webofscience.com/wos/alldb/basic‐search (accessed on June 17, 2023).
